# Description of *Alpheus cedrici* sp. n., a strikingly coloured snapping shrimp (Crustacea, Decapoda, Alpheidae) from Ascension Island, central Atlantic Ocean

**DOI:** 10.3897/zookeys.183.3073

**Published:** 2012-04-19

**Authors:** Arthur Anker, Sammy De Grave

**Affiliations:** 1 Instituto de Ciências do Mar (Labomar), Universidade Federal do Ceará (UFC), Fortaleza, Ceará, Brazil; 2 Oxford University Museum of Natural History, Oxford, United Kingdom

**Keywords:** Caridea, Alpheidae, new species, *Alpheus*, Ascension Island, Atlantic Ocean

## Abstract

*Alpheus cedrici* **sp. n.** is described based on two specimens collected under rocks while scuba diving off the coast of Ascension Island, central Atlantic Ocean. The new species belongs to the *Alpheus macrocheles* (Hailstone, 1835) species complex and appears to be most closely related to the eastern–central Atlantic *Alpheus macrocheles*, the western Atlantic *Alpheus amblyonyx* Chace, 1972, and the eastern Pacific *Alpheus bellimanus* Lockington, 1877 and *Alpheus rectus* Kim & Abele, 1988. However, it differs from all these species by a combination of morphological characters and by a diagnostic and striking colour pattern.

## Introduction

The current knowledge of the alpheid shrimp fauna of the isolated central Atlantic islands St. Helena and Ascension is mainly based on two accounts, Chace (1966) for St. Helena and Manning and Chace (1990) for Ascension. Chace (1966) reported only three alpheid species from St. Helena, viz. *Alpheus macrocheles* (Hailstone, 1835), *Synalpheus fritzmuelleri* Coutière, 1909, and *Metalpheus paragracilis* (Coutiere, 1897). Manning and Chace (1990) reported the same three alpheids from Ascension Island, and in addition *Alpheus bouvieri* A. Milne Edwards, 1878, *Alpheus crockeri* (Armstrong, 1941) [with some doubts], *Alpheus dentipes* Guérin, 1832, *Alpheus holthuisi* Ribeiro, 1964, *Alpheus paracrinitus* Miers, 1881, *Automate dolichognatha* De Man, 1888, *Metalpheus rostratipes* (Pocock, 1890), *Parabetaeus hummelincki* (Schmitt, 1936) [as *Neoalpheopsis euryone* (De Man, 1910)], *Salmoneus setosus* Manning & Chace, 1990, and *Salmoneus teres* Manning & Chace, 1990, resulting in a total of 13 species of Alpheidae known to occur in the Central Atlantic Ocean south of Equator. Most alpheid specimens reported in Chace (1966) and Manning and Chace (1990) were collected in intertidal and shallow subtidal habitats, in tide pools, under rocks, in crevices of rocks and conglomerates of coralline algae, or in buoy fouling.

In April 2008, while scuba diving in English Bay, Ascension Island, one of us (SDG) collected two strikingly coloured snapping shrimps, by flipping rocks at a depth range of 10–15 m. A closer examination of these specimens revealed that they belong to a hitherto unnamed species of *Alpheus* Fabricius, 1798. This species is herewith described as new. Type material is deposited in the collections of the Oxford University Museum of Natural History, Oxford, the United Kingdom (OUMNH.ZC). Abbreviations used in the text: cl, carapace length (measured from the tip of the rostrum to the posterior margin of the carapace); Mxp, maxilliped; P, pereiopod; CA, central Atlantic; EA, eastern Atlantic; WA, western Atlantic; EP eastern Pacific.

## Systematics

### Family Alpheidae Rafinesque, 1815

**Genus *Alpheus* Fabricius, 1798**

#### Alpheus cedrici sp. n.

urn:lsid:zoobank.org:act:7887B4BB-52D9-4329-A8BC-F32D8FA19FFC

http://species-id.net/wiki/Alpheus_cedrici

Figs 1 [Fig F2] 3

##### Material examined.

Holotype: male, cl 10.1 mm, OUMNH.ZC.2008-11-0017, Ascension Island, west side of English Bay, 07°53.675'S, 014°22.999'W, depth 10 m, under rocks, leg S. & H. De Grave, 16.04.2008. Paratype: ovigerous female, cl 11.8 mm, OUMNH.ZC.2008-11-0018, Ascension Island, west side of English Bay, 07°53.675'S 014°22.999'W, depth 15 m, under rocks, leg. S. & H. De Grave, 17.04.2008.

##### Comparative material examined.

*Alpheus macrocheles* (Hailstone, 1835): 1 male, cl 9.0 mm, OUMNH.ZC.2003-36-0002, Madeira, Canico, depth 20 m, leg. P. Wirtz, 02.11.2003. *Alpheus amblyonyx* Chace, 1972: 1 male, cl 5.6 mm, OUMNH.ZC.2011-03-0070, Panama, Isla Grande, in coral rubble, 1–1.5 m, leg. A. Anker, 09.12.2006.

##### Diagnosis.

Frontal margin of carapace with rostrum slightly flattened dorsally, tapering distally, with acute tip, much longer than wide, reaching half-length of first article of antennular peduncle; rostral carina not distinct; orbital teeth in marginal position, small, acute distally, shorter than rostrum; margin between orbital teeth and rostrum broadly V-shaped; rostro-orbital process present; pterygostomial angle rounded; antennular peduncle with stylocerite not reaching distal margin of first article, with acute tip; second article about 2.5 times as long as wide; antenna with basicerite terminating in sharp distoventral tooth; carpocerite slightly exceeding both scaphocerite and antennular peduncle; scaphocerite with well-developed blade, shallowly concave lateral margin and large, stout distolateral tooth, latter reaching far beyond distal margin of blade; male minor cheliped with ventromesial margin of merus ending in small, acute distomesial tooth, and with minute spiniform setae; palm strongly compressed, with sculpture on both lateral and mesial surfaces, consisting of low crests ending in sharp teeth distally; lateroventral surface with distinct, rounded shoulder; pollex shallowly excavated on cutting edge; dactylus somewhat flattened and twisted laterally, not conspicuously broadened, only slightly convex dorsally; male and female major chelipeds similar in shape and in proportions; ventromesial surface of ischium with small spiniform seta; ventromesial margin of merus straight, ending in stout, sharp distomesial tooth, and with small, widely spaced spiniform setae; palm somewhat compressed, with strong sculpture on lateral and mesial surfaces, consisting of low crests ending in sharp teeth distally; lateroventral surface with rounded, smooth, non-projecting shoulder adjacent to deep notch, latter continuing transversely to shallow groove on mesial surface; dorsal margin with subcylindrical elevation ending in large adhesive disk distally; distomesial surface with transversally deeply notched crest ending in sharp tooth; pollex shorter than dactylus, somewhat twisted and shallowly depressed laterally, cutting edge bluntly projecting laterally; dactylus flattened, twisted laterally, convex dorsally, bulbous distally, plunger reduced to broad, low tooth; second pereiopod with five-articulated carpus, ratio of articles approximately equal to 4 : 2 : 1 : 1.5 : 2; third and fourth pereiopods similar; ischium armed with spiniform seta on ventrolateral surface; merus about five times as long as wide, without distoventral tooth; propodus with about eight spiniform setae along ventral margin and additional pair of spiniform setae close to propodo-dactylar articulation; dactylus about 0.4 length of propodus, simple, conical, faintly curved, with acute tip; pleopods with protopods furnished with spiniform setae on lateral margin, some inserted in pairs; male second pleopod with appendix masculina subequal in length to appendix interna, not reaching distal margin of endopod; uropodal exopod with sinuous diaeresis and small distolateral spiniform seta; uropodal endopod with row of small spiniform setae along distolateral margin; telson subrectangular, tapering posteriorly, about twice as long as wide at base; dorsal surface with two pairs of strong spiniform setae, first pair anterior to telson mid-length, second pair at about 0.7 telson length; posterior margin broadly convex, with two pairs of posterolateral spiniform setae, mesial about twice as long as lateral; anal tubercles well developed; gill–exopod formula typical for genus.

**Figure 1.**
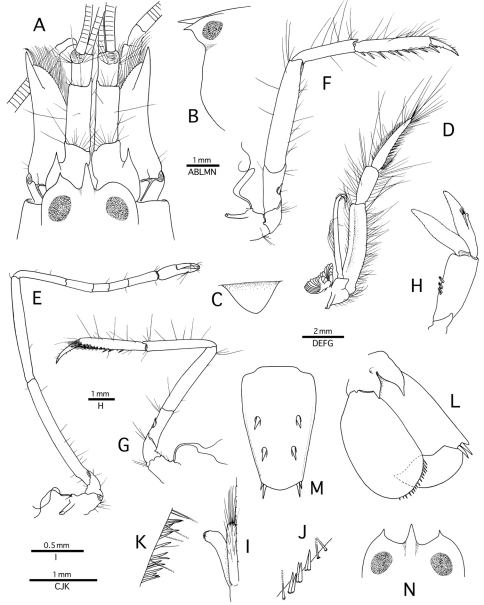
*Alpheus cedrici* sp. n. **A–M** holotype, male from Ascension Island (OUMNH.ZC. 2008-11-0017); **N** paratype, female, same locality (OUMNH.ZC. 2008-11-0018). **A** frontal region, dorsal **B** anterior carapace, lateral **C** tooth of ventromesial carina of first article of antennular peduncle, lateral **D** third maxilliped, lateral **E** second pereiopod, lateral **F** third pereiopod, lateral **G** fifth pereiopod, lateral **H** second pleopod, lateral **I** same, appendix masculina and appendix interna, mesial **J** same, detail of spiniform setae on protopod, lateral **K** third pleopod, detail of spiniform setae on protopod, mesial **L** uropod, dorsal **M** telson, dorsal **N** anterior carapace, dorsal.

**Figure 2. F2:**
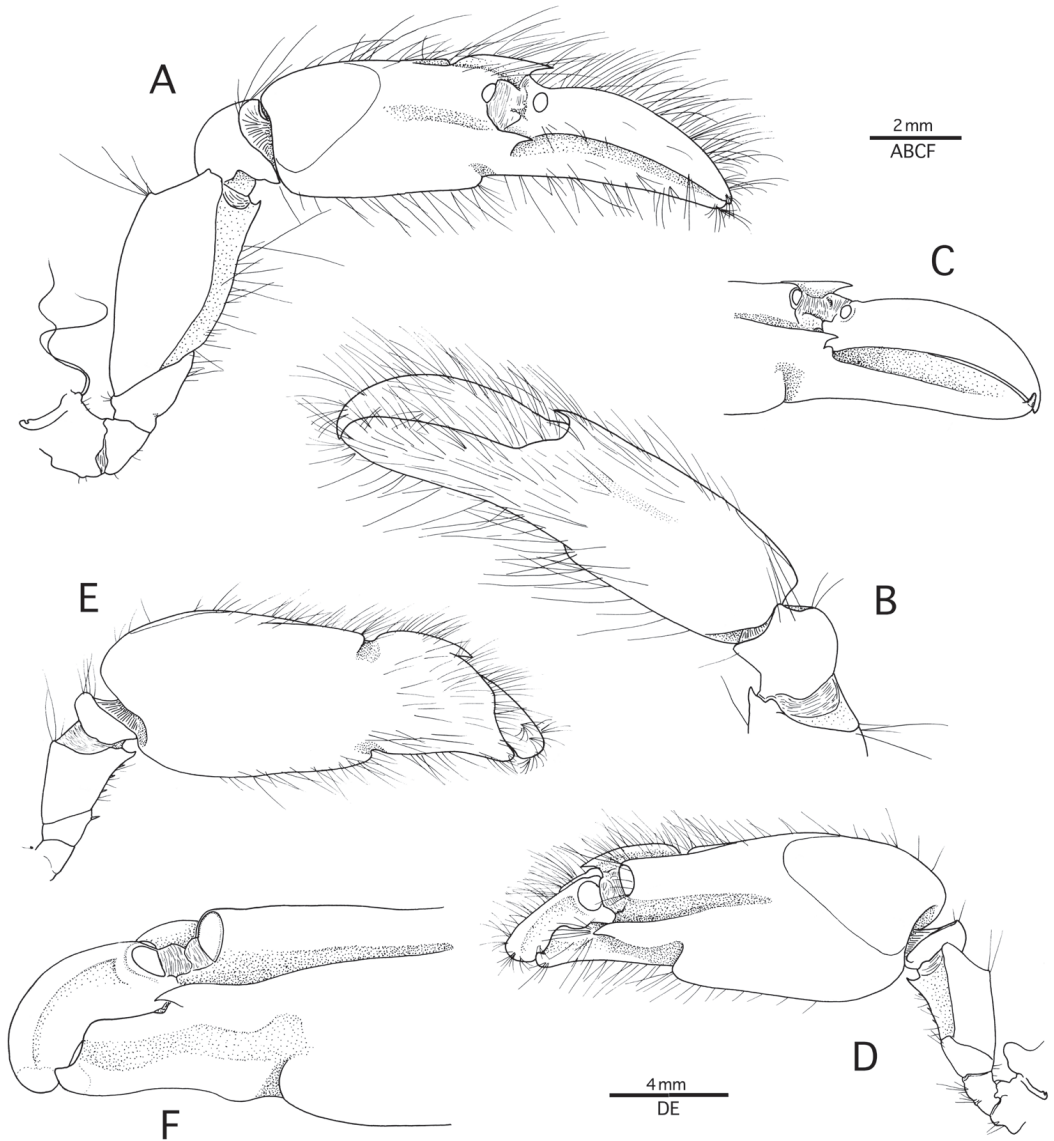
*Alpheus cedrici* sp. n. Holotype, male from Ascension Island (OUMNH.ZC. 2008-11-0017). **A** minor (right) cheliped, lateral **B** minor (right) chela and carpus, mesial **C** same, distal palm and fingers, lateral, setae omitted **D** major (left) cheliped, lateral **E** major (right) cheliped, mesial **F** same, distal palm and fingers, lateral, setae omitted.

**Figure 3.**
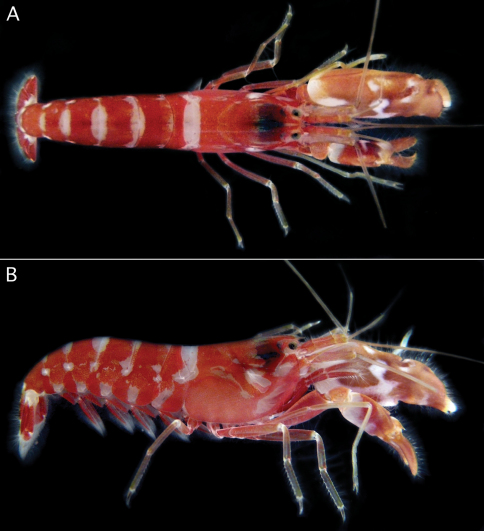
*Alpheus cedrici* sp. n. Holotype, male from Ascension Island (OUMNH.ZC. 2008-11-0017). **A** dorsal view **B** lateral view (photographs by S. De Grave).

##### Description.

Body stout, laterally not compressed. Carapace glabrous; frontal margin with well-developed rostrum and orbital teeth; rostrum slightly flattened dorsally, tapering distally, with acute tip, much longer than wide; lateral margins without setae; tip reaching half-length of first article of antennular peduncle; rostral carina not distinct; orbital teeth in marginal position, relatively small, acute distally, shorter than rostrum (note: right orbital hood atypical, i.e. without tooth in male); margin between orbital teeth and rostrum broadly V-shaped; orbital hoods moderately swollen, enclosing eyes from all sides (Fig. 1A, B); rostro-orbital process present. Pterygostomial angle rounded, not protruding anteriorly (Fig. 1B); cardiac notch deep. Abdominal somites with posteroventral margins broadly rounded, fifth slightly more angular; sixth somite without articulated flap, bluntly projecting posteriorly.

Eyes with well-developed corneas; anteromesial margin bluntly protruding. Ocellar beak projecting, acute, visible in lateral view. Epistomial sclerites not acutely projecting.

Antennule with moderately slender peduncle; stylocerite not reaching distal margin of first article, with acute tip; ventromesial carina with large, subtriangular tooth as illustrated (Fig. 1C); second article much longer than dorsally visible portion of first article, about 2.5 times as long as wide (Fig. 1A); lateral flagellum with groups of aesthetascs starting from 12^th^ article. Antenna with basicerite terminating in sharp distoventral tooth; carpocerite slightly exceeding both scaphocerite and antennular peduncle; scaphocerite with shallowly concave lateral margin and large, stout distolateral tooth, latter reaching far beyond distal margin of blade (Fig. 1A).

Mouthparts (not dissected) not specific in external view. Third maxilliped rather slender; coxa with lateral plate somewhat truncate distally; exopod long, overreaching distal margin of antepenultimate article; antepenultimate article somewhat flattened, ventral margin densely setose; penultimate article no more than three times as long as greatest width, distally slightly widening, very setose; ultimate article slender, tapering distally, with rows of serrulate setae and long, simple setae, tip unarmed (Fig. 1D).

Male minor cheliped with short, stout ischium; merus broad, subtriangular in cross-section; ventrolateral margin smooth; ventromesial margin straight, ending in small, acute distomesial tooth, and with four minute spiniform setae roughly equidistantly spaced along 0.6–0.7 of merus margin, and with tips falling just short of margin (therefore invisible in lateral view); distodorsal angle blunt; carpus rounded, cup-shaped; chela strongly compressed, with palm sculptured distally; lateral surface with low crest starting at about mid-length of palm and ending in a sharp distolateral tooth; ventral margin with blunt, non-protruding shoulder and adjacent deep notch, latter continuing transversely forming a shallow depression on mesial surface; dorsal margin with subcylindrical elevation ending distally in small adhesive disk; distomesial surface with crest ending in stout sharp tooth; fingers as long as palm; pollex shallowly excavated on cutting edge; dactylus somewhat flattened and twisted laterally, slightly convex dorsally, proximally with small adhesive disk (Fig. 2A–C). Female minor cheliped unknown (missing in the paratype).

Male major cheliped with short, stout ischium, ventromesial surface with small spiniform seta; merus stout, short, broad, subtriangular in cross-section; ventrolateral margin smooth; ventromesial margin straight, ending in stout, sharp distomesial tooth, and with small, widely spaced spiniform setae; dorsal margin ending bluntly distally; carpus very short, cup-shaped; chela somewhat compressed; palm strongly sculptured; lateral surface with low crest starting at about 0.6 length of palm and ending in sharp distolateral tooth; ventral margin with rounded, smooth, non-projecting shoulder adjacent to deep notch, latter continuing transversely to shallow groove on mesial surface; dorsal margin with subcylindrical elevation ending in large adhesive disk distally; distomesial surface with transversally deeply notched crest ending in sharp tooth; pollex shorter than dactylus, somewhat twisted and shallowly depressed laterally, cutting edge bluntly projecting laterally; dactylus flattened, twisted laterally, convex dorsally, bulbous distally, plunger reduced to broad, low tooth (Fig. 2D–F). Female major cheliped generally similar in shape and proportions to male major cheliped.

Second pereiopod elongate, slender; ischium slightly longer than merus; carpus with five articles with ratio approximately equal to 4 : 2 : 1 : 1.5 : 2; chela simple, fingers with scarce tufts of setae (Fig. 1E). Third and fourth pereiopods generally similar, moderately slender; third pereiopod with ischium armed with spiniform seta on ventrolateral surface; merus about five times as long as wide, without distoventral tooth; carpus more slender and about half as long as merus; propodus longer than carpus, with eight or so robust spiniform setae along ventral margin and one pair of longer spiniform setae adjacent to dactylus; dactylus about 0.4 length of propodus, simple, conical, faintly curved, with acute tip (Fig. 1F). Fifth pereiopod much more slender than third and fourth pereiopods; merus only slightly longer than carpus; ischium with spiniform seta; propodus with some spiniform setae along ventral margin and well-developed setal brush distolaterally (Fig. 1G).

Pleopods with protopods furnished with spiniform setae on lateral margin, some inserted in pairs (Fig. 1H, J, K), first pleopod with small endopod furnished with setae, male second pleopod with appendix masculina subequal in length to appendix interna, not reaching distal margin of endopod, furnished with numerous stiff setae (Fig. 1I); female second pleopod with appendix interna only. Uropod with lateral lobe of protopod ending in large, acute tooth; diaeresis sinuous, with blunt tooth adjacent to one (occasionally two) stout distolateral spiniform seta(e); endopod with row of small spiniform setae along distolateral margin (Fig. 1L).

Telson subrectangular, tapering towards posterior margin, about twice as long as wide at base; lateral margins slightly convex; dorsal surface with two pairs of strong spiniform setae inserted at some distance from lateral margin, first pair anterior to telson mid-length, second pair at about 0.7 telson length; posterior margin about 0.6 length of anterior margin, broadly convex, with two pairs of posterolateral spiniform setae, mesial about twice as long as lateral (Fig. 1M); anal tubercles well developed.

Gill–exopod formula typical for *Alpheus*: five pleurobranchs (above P1–5), one arthrobranch (Mxp3), two lobe-shaped epipods (Mxp1–2), five mastigobranchs (Mxp3, P1–4), five setobranchs (P1–5); three exopods (Mxp1–3).

##### Size.

*Alpheus cedrici* sp. n. is a medium-sized species of *Alpheus*, with 10.1 mm cl for the male, and 11.8 mm for the ovigerous female.

##### Colour.

Body ground colour bright red or red–orange; carapace mostly red with transverse white band along posterior margin and several colourless or whitish areas on flanks; abdomen mostly red with transverse, more or less oval-shaped, white bands, latter mainly dorsal and not extending to ventral margins of pleura, additional colourless or whitish patches present near ventral margin of each pleuron; major chelae orange–brown marbled with pale yellow on mesial side, and with a distinct, somewhat zigzag-shaped, transverse, white band on palm, extending ventrally and posteriorly; dactylus pale brown with white tip; minor chela similar to major chela, orange–brown with transverse white bands on palm, a broader, more diffuse distal band, and smaller, well-delimited, V-shaped, proximal band; second to fifth pereiopods pale reddish to yellowish, with white articulations; pleopods red; uropods and telson mostly red except for white uropodal protopods and most proximal portion of telson (Fig. 3).

##### Etymology.

Named after our friend and colleague, Dr. Cedric d’Udekem d’Acoz, in recognition of his important contribution to the taxonomy of caridean shrimp and other decapods, particularly in the Atlantic Ocean.

##### Habitat.

Both specimens were collected by lifting large, shallowly buried rocks on a fine sandy substrate at depths between 10 and 15 m, where the marginal boulder talus meets the sand.  Other decapods obtained in the same habitat were the alpheids *Automate* cf. *dolichognatha*, *Alpheus holthuisi*, *Metalpheus paragracilis*, and the axiid *Axiopsis* cf. *serratifrons* (A. Milne-Edwards, 1873).

##### Type locality.

English Bay, Ascension Island, central Atlantic Ocean.

##### Distribution.

Central Atlantic Ocean: currently known only from the type locality.

##### Remarks.

*Alpheus cedrici* sp. n. belongs to the monophyletic *Alpheus macrocheles* species group, which is comprised of about 30 species worldwide, all sharing a unique sculpture of the major chela (e.g. Coutière 1905; De Man 1911; Crosnier and Forest 1966; Banner 1953; Banner and Banner 1982; Kim and Abele 1988). Within the *Alpheus macrocheles* species group, *Alpheus cedrici* sp. n. belongs to the *Alpheus macrocheles* species complex, characterised by the presence of acuminate orbital teeth on the frontal margin of the carapace; the major cheliped bearing a well-developed ventral notch and a dorsomesial notch or constriction; and the third and fourth pereiopods (P3–4) with unarmed meri and simple or minutely biunguiculate (not conspicuously biunguiculate) dactyli. The majority of species in the *Alpheus macrocheles* complex are found in the Atlantic Ocean: *Alpheus macrocheles* (EA, CA), *Alpheus platydactylus* Coutière, 1897 (EA), *Alpheus amblyonyx* Chace 1972 (WA), *Alpheus lentiginosus* Anker & Nizinski, 2011 (WA), *Alpheus puapeba* Christoffersen, 1979 (WA), *Alpheus pouang* Christoffersen, 1979 (WA), and *Alpheus cedrici* sp. n. (CA). Two species are distributed in the eastern Pacific: *Alpheus bellimanus* Lockington, 1877 (EP), and *Alpheus rectus* Kim & Abele, 1988 (EP). Finally, only one Indo-West Pacific species presents the above combination of characters: *Alpheus albatrossae* (Banner, 1953). All these species are contrasted and compared with the new species below, in order of geographical proximity.

**Figure 4.**
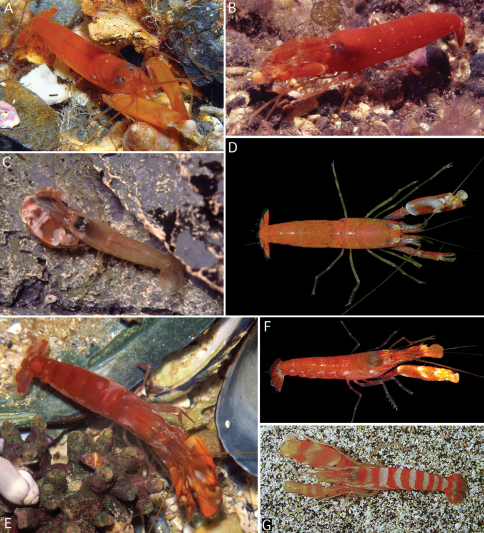
Colour patterns of some species of the *Alpheus macrocheles* (Hailstone, 1835) complex. **A** *Alpheus macrocheles* from Cadaques, Mediterranean coast of Spain **B** *Alpheus macrocheles* from Madeira **C** *Alpheus amblyonyx* Chace, 1972 from Guadeloupe **D** *Alpheus amblyonyx* from Isla Grande, Panama **E** *Alpheus bellimanus* Lockington, 1877 from Santa Barbara, California **F** *Alpheus bellimanus* from Galapagos **G** *Alpheus* sp. ? *rectus* Kim & Abele, 1988, from the Gulf of California. Photographic credits: A, Josep Lluis Peralta; B, Peter Wirtz; C, Frédéric Fasquel; D, Arthur Anker; E, Gregory Jensen; F, Todd Zimmerman (courtesy of Cleveland Hickman); G, Alex Kerstitch (from Kerstitch 1988, courtesy of A. Kerstitch).

*Alpheus macrocheles* is a well-known, mostly shallow-water species (0–50 m, exceptionally to 185 m), ranging in the eastern Atlantic from the British Isles and Mediterranean Sea south to Gabon, and extending to the Central Atlantic islands of Ascension and St. Helena (Holthuis 1951; Crosnier and Forest 1966; Chace 1966; Manning and Chace 1990). *Alpheus cedrici* sp. n. can be separated from *Alpheus macrocheles* by the presence of a row of spiniform setae on the protopods of the pleopods (absent in *Alpheus macrocheles*); the scaphocerite with a better developed blade (cf. Fig. 1A and Crosnier and Forest 1966, fig. 2a); and the male minor chela being more slender and with the dactylus less expanded and less arched dorsally (cf. Figs. 2A–C and Crosnier and Forest 1966, fig. 2c). The two species also differ in their colour patterns: the white bands and patches on the abdomen of *Alpheus cedrici* sp. n. are contrasting with the mostly uniform deep-red to bright or pale orange abdomen of *Alpheus macrocheles* (Fig. 4A, B). All records of *Alpheus macrocheles* from the western Atlantic, e.g. records from Brazil (Ramos-Porto 1979; Guterres et al. 2005), have to be treated with some caution as they may refer to *Alpheus amblyonyx* or other species of the *Alpheus macrocheles* complex.

*Alpheus platydactylus* is a poorly known deep-water species (50–600 m) restricted to the northeastern Atlantic (Mediterranean Sea to the Azores and Cape Verde). *Alpheus cedrici* sp. n. can be easily distinguished from *Alpheus platydactylus* by the much stouter, shorter antennular peduncles; the broader scaphocerite; the smooth ventral margin of the major chela palm (vs. rugose in *Alpheus platydactylus*); the stouter minor chela, with the relatively shorter fingers; and the less slender second pereiopod (cf. Figs. 1, 2 and Crosnier and Forest 1966, fig. 2e–h).

*Alpheus amblyonyx*, a species widespread in the western Atlantic from the Gulf of Mexico to Brazil (Chace 1972; Christoffersen 1998), is generally very similar to *Alpheus macrocheles*. Chace (1972) separated *Alpheus amblyonyx* from *Alpheus macrocheles* mainly on the basis of four characters: (1) the more prominent rostrum; (2) the transverse notch on the mesiodorsal surface of the major chela palm broader and less sharply defined; (3) the major chela dactylus more strikingly bulbous distally; and (4) the minor chela dactylus without a high dorsal crest. With the exception of the last feature, *Alpheus cedrici* sp. n. can be separated from *Alpheus amblyonyx* using the same criteria as from *Alpheus macrocheles* (see above). The colour pattern of *Alpheus amblyonyx* (Fig. 4C, D) is much more similar to the colour pattern of *Alpheus macrocheles* (Fig. 4A, B) than to that of *Alpheus cedrici*. sp. n. (Fig. 3).

*Alpheus pouang* and *Alpheus puapeba* are two deep-water species presently known only from the southwestern Atlantic, off southern Brazil and Uruguay, at depth ranges of 120–268 m and 45–175 m, respectively (Christoffersen 1979, 1998). *Alpheus cedrici* sp. n. can be distinguished from *Alpheus pouang* by the anterior margin of the carapace between the rostrum and the orbital teeth being shallowly and broadly concave (vs. much more deeply incised in *Alpheus pouang*), and the minor chela with a non-protruding ventral shoulder and a distinctly less flattened and dorsally arched dactylus (cf. Figs. 1–2 and Christoffersen 1979, figs. 14–15). The new species differs even more from *Alpheus puapeba*, for example, by the much shorter antennular peduncles and the less elongate, more swollen major chela (cf. Figs. 1–2 and Christoffersen 1979, figs. 16–17). In addition, the pleopodal protopods of both *Alpheus pouang* and *Alpheus puapeba* are not armed with rows of spiniform setae (Christoffersen 1979, figs. 15r, 17d, 18f), as is the case of *Alpheus cedrici* sp. n. (Fig. 1J).

*Alpheus lentiginosus* is another deep-water western Atlantic species presently known only from the northern Gulf of Mexico, at depths of 336–438 m (Anker and Nizinski 2011). *Alpheus cedrici* sp. n. can be separated from *Alpheus lentiginosus* by the less expanded, dorsally non-arched dactylus of the minor chela; the less slender third to fifth pereiopods, with simple, conical dactyli (vs. with a minute accessory unguis on the flexor margin in *Alpheus lentiginosus*); and the presence of spiniform setae on the pleopodal protopods (absent in *Alpheus lentiginosus*) (cf. Figs 1–2 and Anker and Nizinski 2011, figs. 1–2). The colour patterns of *Alpheus cedrici* sp. n. and *Alpheus lentiginosus* are different as well (cf. Fig. 3 and Anker and Nizinski 2011, fig. 3).

The two eastern Pacific species of the *Alpheus macrocheles* complex, *Alpheus bellimanus* and *Alpheus rectus*, are both morphologically very close to *Alpheus cedrici* sp. n. *Alpheus bellimanus* is a relatively common species with a very wide depth range (0–300 m), and also with a wide geographic range, from California via Mexico, Panama and Galapagos to northern Chile (Kim and Abele 1988). *Alpheus rectus* is a much less common species from moderately deep-water (55–73 m); it is currently known only from the type locality in Panama and one locality in southern Baja California (Kim and Abele 1988). *Alpheus cedrici* sp. n. shares with *Alpheus bellimanus* the presence of spiniform setae on the protopods of pleopods. The two species also have very similar frontal margins of the carapace, antennules and antennae, major chelipeds, and walking legs. However, *Alpheus cedrici* sp. n. can be separated from *Alpheus bellimanus* by the non-protruding ventral shoulder of the male minor chela (vs. protruding in *Alpheus bellimanus*); the less expanded, dorsally non-arched dactylus of the male minor chela (vs. more expanded and dorsally strongly convex in *Alpheus bellimanus*); and the anteriorly rounded tooth on the ventromesial carina of the first article of the antennular peduncle (vs. with a subacute tooth in *Alpheus bellimanus*) (cf. Figs. 1–2 and Kim and Abele 1988, fig. 5). The colour pattern of *Alpheus bellimanus* (Fig. 4E, F) resembles more the uniform colour patterns of *Alpheus macrocheles* (Fig. 4A, B) and *Alpheus amblyonyx* (Fig. 4C, D) than the distinctly banded colour pattern of *Alpheus cedrici* sp. n. (Fig. 3).

The new species from Ascension also differs from *Alpheus rectus*, for example, by the less rectangular general shape of the major chela, with the ventral shoulder of the palm broadly rounded, not protruding anteriorly (vs. bluntly projecting in *Alpheus rectus*); and the anteriorly rounded tooth on the ventromesial carina of the first article of the antennular peduncle (vs. with a small point in *Alpheus rectus*). The colour pattern of *Alpheus rectus* remains unconfirmed. However, a colour photograph of a snapping shrimp erroneously identified as “*Alpheus paracrinitus*” in Kerstitch (1988) matches *Alpheus rectus*, especially in the shape of the major and minor chelipeds. The colour pattern of this individual (*Alpheus* sp. ? *rectus* in Fig. 4G), although characterised by a conspicuous transversal orange-white banding, is different from that of *Alpheus cedrici* sp. n., especially in the clearly banded carapace and the abdominal bands extending ventrally to the pleural margins (cf. Figs. 3, 4G).

All other species of the *Alpheus macrocheles* group present in the western and eastern Atlantic and in the eastern Pacific differ more markedly from *Alpheus cedrici* sp. n. (see Coutière 1910; Armstrong 1940, 1941; Holthuis 1951; Crosnier and Forest 1966; Chace 1972; Wicksten and Méndez 1981; Kim and Abele 1988; Wicksten and McClure 2003; Anker et al. 2008). The shape of the frontal margin of the carapace separates the new species from *Alpheus inca* Wicksten & Méndez, 1981, *Alpheus grahami* Abele, 1975, *Alpheus cylindricus* Kingsley, 1878, *Alpheus vanderbilti* Boone, 1930, *Alpheus clamator* Lockington, 1877, *Alpheus peasei* (Armstrong, 1940), *Alpheus dentipes* Guérin, 1832 and *Alpheus candei* Guérin-Méneville, 1855). The shape of the major cheliped separates the new species from *Alpheus crockeri* (Armstrong, 1941), *Alpheus hortensis* Wicksten & McClure, 2003, *Alpheus grahami*, *Alpheus cylindricus* and *Alpheus vanderbilti*. The shape of the minor cheliped separates the new species from *Alpheus hoonsooi* Kim & Abele, 1988, *Alpheus crockeri*, *Alpheus hortensis*, *Alpheus grahami*, *Alpheus cylindricus* and *Alpheus vanderbilti*. The presence of a distinct distoventral tooth on the merus of the third and fourth pereiopod in *Alpheus hoonsooi*, *Alpheus clamator*, *Alpheus peasei* and *Alpheus dentipes* separates these species from *Alpheus cedrici* sp. n. Finally, the strongly biunguiculate dactylus of the third to fifth pereiopods in *Alpheus clamator*, *Alpheus peasei*, *Alpheus dentipes* and *Alpheus candei* separates these species from *Alpheus cedrici* sp. n.

*Alpheus cedrici* sp. n. can be separated from the Indo-West Pacific *Alpheus albatrossae* by the presence of a distinct shoulder on the ventrolateral surface of the minor chela palm (absent in *Alpheus albatrossae*); the stouter fingers of the minor chela; and the absence of a small unguis on the dorsal margin of the dactylus of the third to fifth pereiopods (present in *Alpheus albatrossae*) (cf. Figs. 1, 2 and Banner, 1953, fig. 18). None of the other Indo-West Pacific species of the *Alpheus macrocheles* group appears to be closely related to *Alpheus cedrici* sp. n. (e.g. Coutière 1905; De Man 1911; Banner 1953; Kensley 1969; Banner and Banner 1982; Burukovsky 1990).

## Supplementary Material

XML Treatment for Alpheus cedrici
